# Control of Canine Visceral Leishmaniasis: A Success Case Based on Deltamethrin 4% Collars

**DOI:** 10.3390/epidemiologia2040035

**Published:** 2021-10-14

**Authors:** Vera Lucia Fonseca de Camargo-Neves, Eliana Bravo Calemes, Lilian Aparecida Colebrusco Rodas, Fredy Galvis-Ovallos, Luis Jacintho da Silva

**Affiliations:** 1 Epidemiology Department, Superintendence for Endemic Diseases Control—Sucen, São Paulo 01027-000, Brazil; 2 Regional Laboratory Center, Adolfo Lutz Institute—IAL, Araçatuba 16015-160, Brazil; eliana.calemes@globo.com; 3 Araçatuba Rgional Center, Superintendence for Endemic Diseases Control—Sucen, Araçatuba 16015-160, Brazil; colerodas@gmail.com; 4 Epidemiology Department, School of Health Public, University of São Paulo, São Paulo 01246-904, Brazil; galvisfregao@gmail.com

**Keywords:** canine visceral leishmaniasis, collar impregnated, control measures, logistic models, associate factors, survival probability

## Abstract

The effect of employing collars impregnated with deltamethrin 4% (DM4) to control canine visceral leishmaniasis (CVL) was evaluated. as were the individual factors associated with this infection. A cohort study that included household dogs was conducted between 2002 and 2006. The presence of pathognomonic signals, peridomiciliary sleep habits and breed were the main factors associated with the infection. The use of DM4 collars contributed to the reduction of CVL with an effectiveness of 66%, and the dogs’ survival rate was greater than 90% at 50 months. In conclusion, the adoption of DM4 collars reduced the number of euthanized canines and in the incidence of CVL, and this reduction was sustained for one year after discontinuing the use of the collar.

## 1. Introduction

In Brazil, visceral leishmaniasis (VL) is a zoonosis that mainly affects children under 10 years old and can cause death when undiagnosed and untreated. Although, the highest incidence occurs in children, the mortality impact, in a majority adults and especially the elderly, related to comorbidities, represent a social problem that impacts the economy due to the reduction in the workforce [1].

VL is caused by the protozoan *Leishmania infantum* and transmitted by the sand fly *Lutzomyia longipalpis*. In urban areas of São Paulo state, the domestic dog is the main source of the blood which feeds this sandfly, [2] and the main reservoir for the VL etiologic agent [3]. The disease is potentially fatal in dogs, and those that survive are sources by which the sand fly spreads infection. 

In São Paulo state, canine visceral leishmaniasis (CVL) is an endemic disease that is spreading in urban areas, currently registered in 145 municipalities [4].

Currently, the main control measures adopted in São Paulo to reduce human morbidity and mortality are the rapid diagnosis and adequate treatment of cases, as well as reducting the number of infected hosts and the vector density. Those measures, when well applied, are enough to control VL in human being; however, they have been shown to be inadequate to achieve a significant reduction in the incidence of CVL [5,6] and when are obtained are not sustained over time [7]. Some factors that could explain these limitations, include scarcities in equipment and human resources, low coverage (due to the large areas requiring coverage), identification of infected dogs (due to serologic diagnosis with low sensitivity and specificity, and knowledge about the individual factors contributing to canine infection [8]. Vector control requires extensive human resources to cover the transmission area in an adequate time; in additionally, the insecticide residual power could be limited to no more than two months in the peridomicile [8].

Especially in urban areas, increased VL infection in dogs also increases the risk of VL in humans [8,9,10]. Therefore, the implementation of innovative measures to reduce vector–host contact is required. The use of other control measures, where the dog population is concerned, can help to reduce the incidence. Some studies [11,12,13] that evaluated the application of collars impregnated with DM4 insecticide have found an effective reduction of VL incidence in the human population. The collars are used to individually protect dogs against sand fly bites, reducing the number of infected dogs, due to their insecticidal and repellent effects, and so decreasing the incidence of CVL [14,15]. 

The dog is the main reservoir of *L. infantum* and the main blood-feeding source for the sand fly in urban areas, thus the occurrence of human cases is associated with the presence of canine cases [16,17,18,19]. In this work, we evaluated the use of collars impregnated with 4% deltamethrin insecticide Scalibor^®^ (DM4), and the euthanasia of seropositive dogs according to the norms of the National Program of Surveillance and Control of Visceral Leishmaniasis (NPCVL) for their effect, over time, in controlling canine VL [20]. We also studied the individual factors associated with *L. infantum* infection in the canine population of the urban area of Andradina, SP.

## 2. Materials and Methods

### 2.1. Study Area and Epidemiological Background

**Figure 1:**
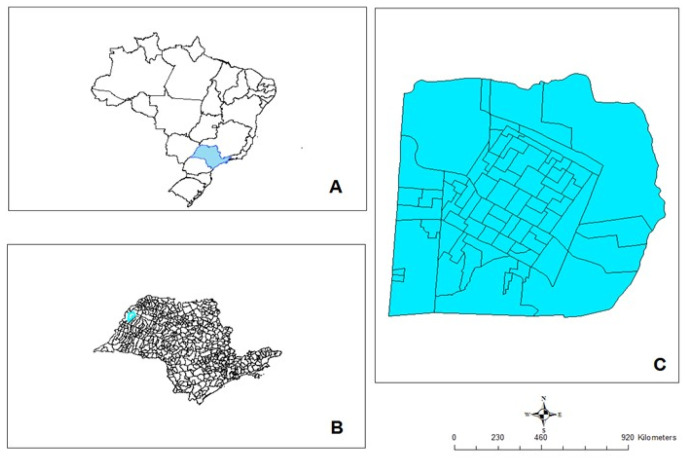
(**A**) Geographic location of the state of São Paulo, Brazil; (**B**) the municipality of Andradina; (**C**) and the urban area of Andradina (study area).

The study was conducted in Andradina municipality, located in the Western region of São Paulo state (20.53° S, 51.22° W) (Figure 1). This municipality has an area of 960.1 Km^2^ and a demographic density of 57 inhabitants/Km^2^. In 2002, its dog population was estimated at 15,600 dogs and its human population at 55,161 inhabitants, of which 93% live in the urban area. Before the year 2002, this area used to be considered free of VL transmission; however, in 2002, the prevalence of CVL reached 11.3% and the incidence of human VL was 38 cases/100,000 inhabitants, with a lethality of 21.1%. 

### 2.2. Study Design

This prospective open cohort study occurred between October 2002 and November 2006. The study included all dogs, domiciled or semi-domiciled, with the consent of their owners to participate in the follow-up, as well as new dogs (born or introduced into the household) in the period between serological evaluations.

**Figure 2:**
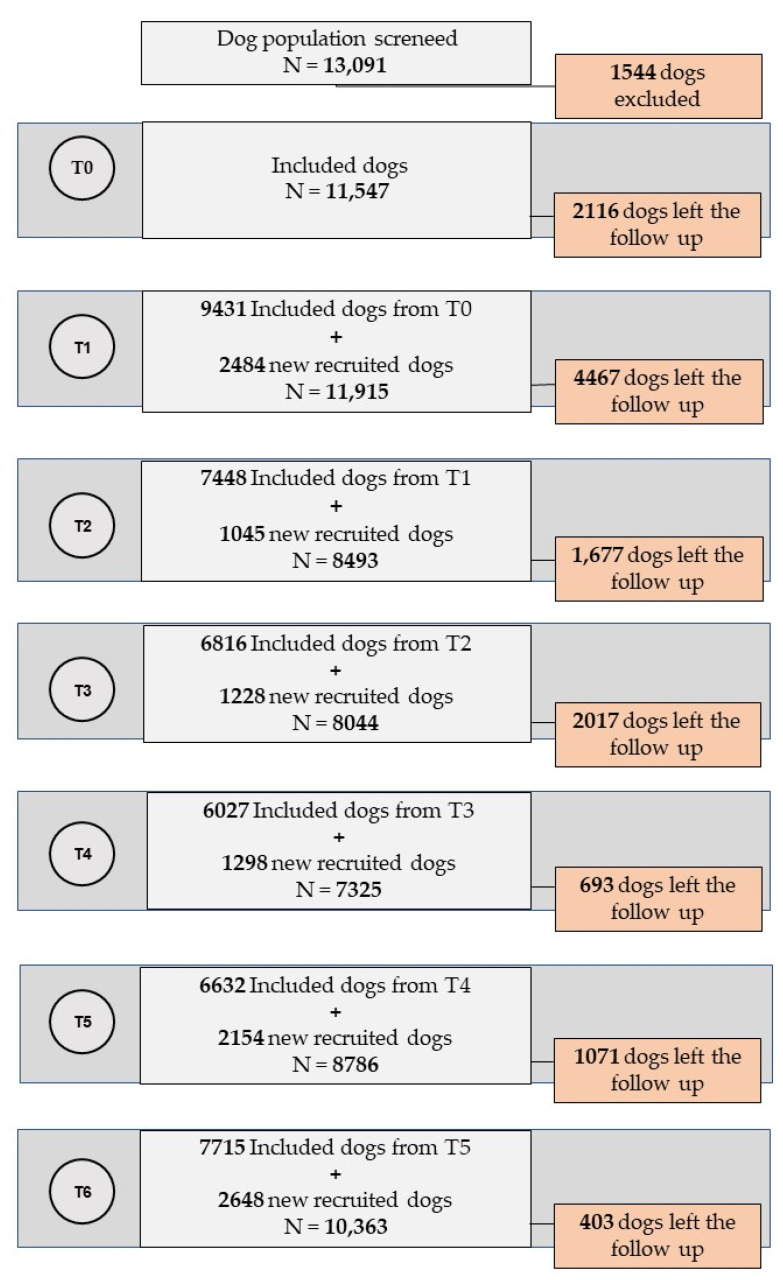
Schematic diagram of the design of the dog cohort, urban area of Andradina, SP, 2002–2006.

The status of canine infection was assessed at each stage (T0 to T6) for 56 months of follow-up. Stage T0 starded in October 2002; T1 in April 2003; T2 in October 2003; T3 in April 2004; T4 in November 2004; T5 in May 2006; and T6 in November 2005. Figure 2 explains the follow up stages for the dog population. 

The collars were introduced at T0 and changed three times, T1 to T3. The dog population was followed for 18 months after the last change of collars (T4 to T6). 

Dogs infected with *L. infantum* were excluded and euthanatized according to the recommendations of the NPCVL of the Brazilian Health Ministry [20]. 

### 2.3. Dog Population Survey

A door-to-door survey was conducted to obtain data about the canine population characteristics: the dog’s age, sex, length and color of hair, weight, corporal size, breed, VL signs, sleeping habit, and owner identification.

### 2.4. Blood Sampling and Serological Survey

To evaluate the infection status of the canine population, total blood samples were collected on filter paper and processed by the regional laboratory of the Adolfo Lutz Institute in Andradina. For the serological survey, an indirect immunofluorescent assay-IFA (Bio-Manguinhos^®^) was employed to measure the antibody levels, which was the official test used by the NPCVL. Samples of dogs presenting a titer of 1:40 or higher were considered positive. 

### 2.5. Fitting Dog Collars

Each dog that met the inclusion criteria received a collar impregnated with Deltamethrin 4%—Scalibor^®^ (Intervet—Schering Plough, Kenilworth, NJ, USA). The collar consisted of a 65 cm strip of white polyvinylchloride (PVC) weighing 25 g which was impregnated with Deltamethrin 40 mg/g. To fit the collar for the first time, each households with dogs that had negative results in the first serological survey (T0) were visisted. The dogs with collars and negative results had changed their collars three times (T1, T2 and T3). The new dogs with negative serological results that introduced until T3 received collars too.

### 2.6. Data Analysis

A database was built in dBase format, and the data were grouped by the follow up stage. A dog’s length of stay in the cohort and the length collar use were estimated based on day when the collar was fitted and the blood sample collected until euthanized/death day or new blood sample was collected.

The characteristics of the dog population were described in percentages. To determine the association between individual factors and the presence of CVL, descriptive analysis obtained in the dog population survey was evaluated by a multiple regression analysis. To evaluate the associated variables, a univariate analysis and the odds ratio values were estimated. Variables with a statistical significance *p* ≤ 0.20 using the “forward-Wald” procedure were included in the final model. 

To evaluate the effect of the impregnated collar on the canine population, we estimated the prevalence of CVL in the first serological survey and the incidence (by 100 dogs) in the other follow-up stages. The survival analysis to evaluate the effect of the collar over time employed a Kaplan-Meier and Cox regression model. The model was adjusted by the individual characteristics of the dogs using those variables with *p* > 0.20 in the univariate analysis. SPSS software v.24 was employed for those analysis. 

To assess the impact of using the impregnated collar over time and space, the canine cases were georeferenced using Batchgeo software (free online version) https://pt.batchgeo.com/ (accessed on 14 September 2020). The addresses that were not found or incomplete but had the block number were plotted according to the coordinates of the corresponding block. The distribution of CVL cases by the construction of surface maps of the point density was analyzed using the ArcGis v. 10.4 software. The product is found by calculating the magnitude per unit area from point resources that fit into a neighborhood around each cell.

## 3. Results

### 3.1. Dynamic of the Cohort 

Between 2002 and 2006, 23,948 dogs were registered, and 13,688 households were visited. The canine density was 1.75 dogs per household. 

In the first half of 2002 (T0), a total of 13,091 dogs living in the urban area were registered. Of these, 1544 (11.7%) were not included in the follow-up because the dogs either (1) died before the first collection stage (T1), (2) were lost when the owners changed their address, (3) were donated or sold, or (4) were reported losts by the owner. Consequently, the initial study population was 11,547 dogs (Figure 2). Of these dogs, 26.8% (1946 dogs) continued to be followed for the 56 months of the study. 

The loss of follow-up represented 55.5% of the total number of dogs included in the study and was higher than the replacement rate (48.5%). The reasons of the loss of follow-up included the euthanasia of infected dogs (19%), spontaneous delivery of the dog by the owner to the Zoonosis Control Center of the municipality (7%), death by CVL or another causes (42%), and various other causes (28%). Of the 3600 positive dogs, 2376 were euthanized, representing 70% euthanasia coverage rate. 

The city registered 1250 refusals by the owners to euthanasia to VL positive dogs and 3730 owners refused to collect blood sampling in at least one of the seven stages. Of the dogs whose death was informed by the owner, 493/5588 (8.9%) were positive for the anti-leishmania antibody. The loss to follow-up due to the owner’s change address or because the dog ran away accounted for 362 dogs, of which 118 had positive VL diagnosis. 

### 3.2. Characteristics of the Dog Population

To characterize the canine population, 22,404 dogs were evaluated in the follow-up, of which 51.9% were males. Their predominant age was between four and eleven months (33.4%). Most of the dogs weighed less than 20 kg (76.5%) and were less than 20 cm tall (45.3%). The most common hair type was short straight hair (73.9%), and black was the most common hair color (38.1%). Mongrel and mixed-breed dogs accounted for 38.9% of the population studied. Most of the dogs slept in the peridomicile (95.9%) and did not exhibit signs of CVL (94.6%). The frequencies observed according to the category of each variable evaluated are presented in Table 1. 

### 3.3. Factors Associated with to Canine Visceral Leishmaniasis

The results of the univariate regression model are presented in Table 1. Taking into account the total population of dogs, positive dogs were observed in all age intervals. CVL positivity and the odds of infection increased with age, (OR = 0.27 at 3 months of age to OR = 1.2 at 6 to 10 years old); however, after 6 years old the infection rate decreased. The highest frequency of canine positivity was in dogs with short and straight hairs (14.4%) and those that weight up to 20 kg (13.4%). The canine positivity was highest in dogs with black hair (7.5%) and mongrel or mixed breed dogs (8.3%). However, the odds of infection were highest in Brazilian mastiff (OR = 3.1), Braque Saint German dog (OR = 2.5), and Labrador retriever (OR = 2.0). Dogs with peridomiciliary sleep habit showed a high frequency of positivity and odds of infection (18.3%, OR = 1.8). Additionally, asymptomatic dogs exhibited higher canine positivity (16.7%); nevertheless, the odds of infection were higher in symptomatic dogs (OR = 2.7). 

Multiple logistic regression analysis included those variables with statistical significance in the univariate analysis. The results obtained in the analysis are presented in Table 2. The factors associated with canine infection were females (OR = 1.10); age interval (OR = 1.54 to 4.45); presence of pathognomonic signals (OR = 2.07); peridomiciliary sleep habit (OR = 2.1), and colored hair (OR = 1.31 to 1.46) compared to white hair. The breeds with the highest association included Cocker Spaniel (OR = 2.08); German Shepherd (OR = 1.92); Brazilian Mastiff (OR = 2.83), Labrador Retriever (OR = 2.13); Pekingese, Shih-tzu and Lhasa group (OR = 1.42); and mongrel or mixed-breed dogs (OR = 1.39). 

### 3.4. Analysis of the Intervention with 4% Deltamethrin Impregnated Collars 

To analyze the intervention, the dynamic of the population in the cohort was followed for 56 months in seven stages (T0–T6). The collars were fit in stages T0 to T3. Of the 22,404 dogs followed up in the seven stages, 16,304 (72.8%) used collars for at least 6 months. The dogs wore the collars for an average of 12.7 months. 

Regarding the time that the dogs wore the collar 16.2% (3,625) wore the collar for 6 months, 18.2% (4076) for 12 months, 20.4% between 18 (3001) and 24 (1560) months, and 18.1% (4046) for 30 months (Table 3). During the entire follow-up of the cohort, 6096 dogs (27.2%) were not collared because they had positive serology results in the serological evaluation at one of the stages.

The analysis by the Cox model on the effect of the DM4 collar use found that the RR increased with age, weight greater than 20 kg, the presence of pathognomies signals, dark hair and Cocker and Labrador breeds (Table 4).

**Figure 3:**
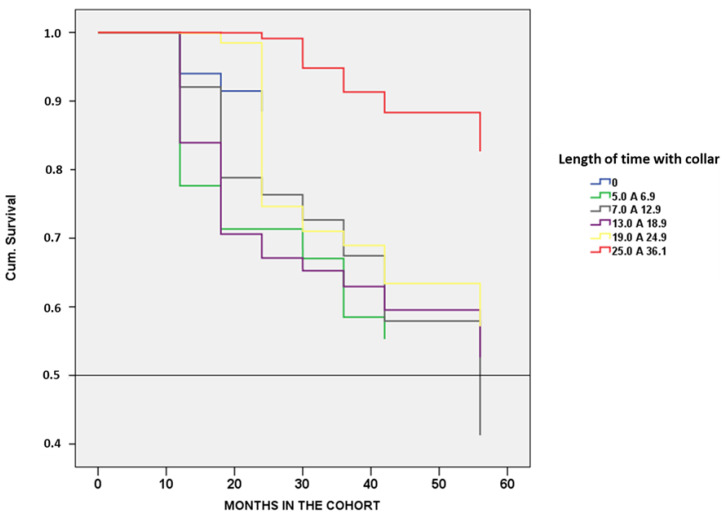
Accumulated survival of the canine population according to the duration that they used the collars.

It was observed that 92.3% of the dogs had a survival higher than 50% with 50 months or more in the cohort when controlled for the independent variables age interval, sex, weight, hair color, sleep habit, breed, and signal presence (Figure 3). From the canine population studied (67.2%), the probability of survival was 75 to 100% after introduction of collars (Table 5).

### 3.5. Prevalence and Incidence of CVL

**Figure 4:**
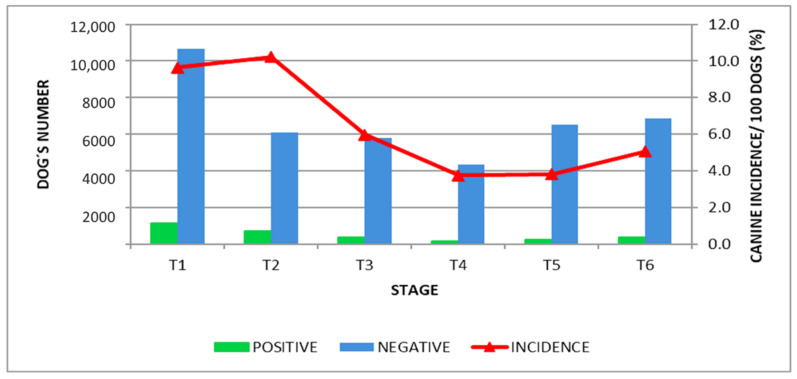
Number of seronegative and seropositive dogs for *Leishmania* antibodies and CLV Incidence Rate.

In the first stage, the estimated prevalence of canine VL was 11.7/100 dogs. A reduction in the incidence of anti-*Leishmania* antibody seropositive dogs was observed after one year of wearing the collar. The reduction in incidence was verified after 18 months of continuous collar use, in the fourth stage to 6.4/100 dogs. The incidence of dogs that were positive for antibodies against *Leishmania* reduced by more than 50% from 10.7/100 dogs to 3.9/100 dogs (Figure 4) in the second and fifth stages, respectively. In the seventh stage, after 12 months without collar use, the incidence increased to 5.3/100 dogs (Figure 4).

**Figure 5:**
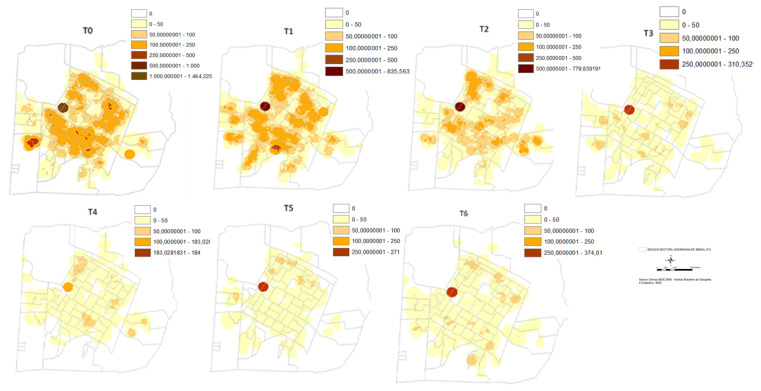
Density maps for canine visceral leishmaniasis positivity over time in the urban area Andradina, SP, 2002–2006.

Figure 5 shows the density maps for canine positivity over time. There was a significant reduction in positivity after the T3 stage in practically all neighborhoods in the city. The hot spots that remained to the east of the city must be carefully analyzed, as this finding may be due to the georeferencing of incomplete addresses, for which the block coordinate pair was considered.

## 4. Discussion

Domestic dogs (*Canis familiaris*) are the main urban host of *L. infantum* due to their high parasite load in the skin and are considered a risk factor for the human infection [21,22]. Independent of their clinical status, dogs can infect sandflies during their entire life, although those with signs related to CVL have a greater chance of infecting sandflies [17,23,24,25]. Therefore, this study aimed to evaluate the population dynamics and factors related to canine infection in a VL endemic area with intense transmission as well as the impact of a control measure (DM4 collar) associated with the canine euthanasia. 

The challenges for the VL control in urban areas were mainly related to the organization, management, and timely implementation of activities by the health services. Currently, the National VL Program conducted by municipalities is affected by the discontinuity of control activities, refusal of dog owners to euthanize infected dogs, low participation of the population in prophylactic activities to reduce the vector density or avoid its presence, and the refusal of the population to apply the insecticide in their houses [8,26,27].

The present study confronted a 21.3% refusal rate for blood sampling. This result is expected in populational studies, with a coverage of about 80%. Furthermore, about 30% of infected dogs were not euthanized because of the owners refusal. This high rate of refusal reflects the population’s lack of knowledge about the risk associated with the presence of positive dogs in households and could make this measure ineffective and difficult to maintain over time. Borges et al. (2006) [9] observed that the presence of dogs in the household increases the odds of human VL infection and that these odds increase directly with the number of household dogs. Thus, our results demonstrated a high risk of human VL infection in the population studied considering the estimated dog density per household (1.75 dog per domicile) and the high proportion (41.2%) of domiciles with two or more dogs. Silva et al. (2012) [10] observed similar results in Teresina, a hyperendemic area in Northeastern Brazil. These authors described that households which had had dogs previously removed through euthanasia had five-time higher odds of having a new infected dog than households that had never had infected dogs. 

The number of dogs lost in the cohort was greater than the replacement of dogs, with 48% of the initial population lost to follow-up. Dispersal of infected dogs to other areas within the locality or to other vulnerable or receptive municipalities contributes to the dissemination of CVL to new areas and hinders its control, as observed by Oliveira et al. (2008) [26]. Considering the canine population dynamic, the reduction in CVL incidence were mainly the result of the implementation of impregnated collars. 

The time between the diagnosis and the dog’s euthanasia affects the result of the control strategies based in euthanasia. Camargo-Neves (2004) [5] observed that the average time between diagnosis and euthanasia was 50 days, suggesting that this time delay is one of the main factors responsible for the maintenance of the canine incidence after four serological surveys, although reduction in human incidence was observed. The present study also found, that the duration between the blood sampling and the diagnostic result was also too long. Therefore, the observed reduction in canine incidence must be related to the fact that the dogs were protected by the collar.

To control human VL, the possibility of maintaining the canine prevalence at constant levels it is a good result that can support the implementation of a control program based on dog euthanasia. However two main factors that limited the success of this control measure are the time between the infection and the seroconversion and the time until removal of the dog. 

The presence of parasites in infected dogs occurs about 10.5 months (varying between 4 to 22 months) before the seroconversion or the clinical manifestation [28,29,30].

VL infected dogs have high parasitism in the skin supporting the infection of female *Lutzomyia longipalpis*. This makes the reduction of the incidence difficult using only a control measure [16,17,18], suggesting the necessity to include new diagnostic methods that detect the host infection [28,31].

Independent of the presence of CVL, the rate of dog turnover was high. Of the dog deaths during the study, 8.8% were infected and 91.2% were due to other causes. In the study, 94.6% of the dogs were asymptomatic and the infection rate was 16.8%. Dogs exhibiting onychogryphosis, weight loss, and hair loss had a 4.1 times greater odds to test positive in the serological test than asymptomatic dogs. The importance of that high rate of asymptomatic dogs is the capacity to remain infected for years or throughout their life without clinical manifestation [17,31] because they could be infection sources [23,24,25,32]. 

The first clinical signs observed in CVL are the popliteal hypertrophy of lymph nodes follow by periorbital or nasal dermatitis, keratoconjunctivitis, alopecia generally together with onychogryphosis, and edema in the legs. Severe cases or death, less frequent in endemic municipalities, are the result of hemorrhage, muscular atrophy, cachexia, anorexia, and loss of weight [33]. Control of infection and manifestation of clinical signs will depend on the type of immune response, parasite inoculate, and nutritional status of the dogs [16,31,34]. Our results are consistent with the finding in other endemic areas, with a higher frequency of onychogryphosis, skin lesions, alopecia, and weight loss [35]. As observed above, the rapid detection of clinical signs in infected dogs is important because they are sources of infection to sandflies. Studies conducted in Europe showed that 100% of symptomatic dogs and 60% of the asymptomatic were infective to the sandfly *Phlebotomus perniciosus* [24,36]. Therefore, the absence of signs in the household population found in this study could be related to the good nutritional conditions of the dogs. Animals with pathognomonic signs of CVL had a 2.7 greater likelihood of having a positive serological result than those without signs. Considering the infectivity of those dogs to the sandflies [17,23,24,25], programs to train health professionals in this aspect and educational programs about the risk of maintaining infected dogs are required. 

The high frequency of asymptomatic dogs reinforces the importance of dogs in maintaining and spreading the parasite, because owners refuse to deliver healthy infected dogs and protect them by sending the dogs to other areas or maintaining them at home, which increases the risk of human infection, as observed in previous studies [35,37].

Courtenay et al. (2002) [38] observed that clinical screening has a high specificity to detect the infectivity of dogs to the sandflies (91.2%) in Brazil. These authors also observed that 62.5% of the female sandflies exposed were infected after exposure to the dogs with more than one symptom when compared with females expose to asymptomatic dogs (21.3%). However, no relation was observed between the clinical score and the number of infected sandflies, suggesting that asymptomatic dogs could act as infection fonts. 

In our study, the canine population was mainly young with 43.2% of the dogs less than one year old and only 11.6% over six years old. However, infection increases with age and attributed risk. In the age range of 6 to 10 years old, the odds of infection were 4.5 times higher than in younger ages, reinforcing the observations of other studies [39,40]. However, different results have been reported in other endemic areas [5,35].

In our study, about 73.8% of the dogs had short straight hair. The infection in this group was 14.4% while in dogs with long hair this value was 2.4%, and the odds of infection were 1.4 and 1.6, respectively. Our results were similar to those obtained in Araçatuba municipality [5], where greater odds of infection were observed in dog with short straight hair. These results are controversial in different studies [32,35,41,42,43,44,45]. However, all of these studies agree with the type of hair (straight), since all the breeds described that showed the highest prevalence were those dogs with straight hair, which was in agreement with our results, and may be related to the access of the vector to the animal’s skin. 

We observed that 56.7% of the dogs had dark hair color (black 38.1%, brown 18.6%), which were also the most likely to have the presence of CVL. In the multiple analysis, all colors were associated with the infection except the spotted color. Twenty-one-breed categories were observed in the study area, with the highest positivity for leishmania in the mongrel or mixed breed group (8.3%). However, the relation between the breed and the CVL infection is a controversial issue and associated genetic factors are unknown. 

Higher odds of infection were observed in medium or large dogs with short straight hair who are mainly associated with protecting the house. According to Dye et al. (1992) [46], CVL is more frequent in working dogs due to their greater exposure to sandfly bites. The force of infection increases with age because more exposition time without protection increase the risk of infection. The categories short and dark color hair, weight, and sleeping location (peridomicile) were associated with increased risk and could be related to the cynophilic behavior of *L. longipalpis* [2]. The multiple analysis evidenced that when grouped by fitness the more susceptible breeds were the Brazilian Mastiff and German Shepherd as well as the companion and guard dogs that include Pekingese/Lhasa/Shih-tzu, Cocker Spaniel, and mongrel or mixed-breed dogs. Our results agree with those reported by França-Silva et al. (2003) [35], who observed that infection is most prevalent in Cocker Spaniel and Boxer breeds. Similarly, Camargo-Neves (2004) [5] observed high prevalence of infection in Cocker Spaniels. 

The use of DM4 impregnated collars contributed to the reduction of the CVL with an effectiveness of 66%. Moreover, the increased survival of dogs was associated with longer times of collar use with survival rate higher than 90% at 50 months when adjusted for the independent variables. The protective effect of the control strategy based on DM4 collars was verified by the reduction of the CVL incidence even year after the last change of collars, suggesting that it was due to the decrease in the infection source of the canine population. Thus, the protective effect of the collar strategy required two years to reduce the area-wide rate of infection and had always been associated with canine euthanasia, and must have the high coverage collar implementation (>70%). The effect of high coverage of the collaring can be seen over time with the reduction of positive CVL density in the entire urban area of Andradina, except for one of the sectors in the east of the municipality. This must be observed carefully due to the flaws in the georeferencing of the addresses because this newer neighborhood many addresses were georeferenced by the block number.

The long effect of collar use is related to its slow-release mechanism. Some experiments have found that the effect of the collar on sandflies occurs after two weeks constant use [14,15,47]. According to those authors, the biting rate could be reduced by 80–96% and the repellency increases with the length of collar use. David et al. (2001) [14] demonstrated that repellent power was 99.3% four weeks after collar use, 100% after 8 to 12 weeks, and 96% between 16 and 22 weeks and, it was observed that the survival rate of *Lu. longipalpis* fed on collared dogs was reduced by 86% [16].

A field study in an endemic area in Iran observed an 80% reduction of *P. papatasi* bites in dogs protected with DM4 collars [47]. A study involving 120 clinically healthy and seronegative dogs observed a protection of 50.8% due to the collars [48]. The use of DM4 collars showed efficacy to reduce the risk of *L. infantum* infection [48,49,50]. Similar results were obtained in studies in Italy [12] and France [51].

The Cox model indicated that when controlled for independent variables, 67.2% of the dogs had a higher than 75% survival considering the entire cohort population. The most important covariables were age, hair color, size, and weight of the dog, as previously observed in the multivariate analysis. 

In Andradina, residual insecticide spraying and environmental management were employed when VL human cases were notified as recommended by the Brazilian control program. In this municipality, the VL incidence was 34.1/100,000 inhabitants in 2002, which reduced to 3.6 cases/100,000 inhabitants in 2004 after the implementation of the DM4 collar program and was zero in 2005. In the third year after removing the collars (2007), the VL incidence reached 5.6 cases/100,000 inhabitants, following the increase in the canine prevalence. Similar results were obtained in Iran where the intervention in the canine population decreased the seroconversion in children [11]. In Italy, Parvizi et al. (2008) [52] also observed that the DM4 collar contributes to the reduction of childhood infection. 

In the same period as our study in municipalities where only the measures recommended by the NPCVL were applied, the incidence rates did not change. In 2002, the municipality of Araçatuba, in which the NPCVL measure has been applied since 1999, had an incidence coefficient of 16.8 cases/100,000 inhabitants, while in the study area it was 23.3 cases/100,000 inhabitants. After two years of the implementation of DM4 collars, the incidence ratio was 3.6 cases/100,000 inhabitants while in Araçatuba it was 21 cases/100,000 inhabitants. Recent studies have demonstrated that the use of this strategy impacts VL transmission. Sevá et al. (2016) [53] employed a mathematical model to demonstrated that impregnated collars reduce human and canine prevalence of VL. Recently, Yimam and Monhebali (2020) [54] elucidated in a meta-analysis study that the use of DM4 could reduce VL incidence. 

However, the use of this measure in a National Program must consider the logistics of its application including the organization of the surveys to change the collars, the storage of the collars, and control the expiration date, and a strategy to replace lost collars and reinforce owner’s adherence. Other strategies that use pyrethroids in Spot-on, Pour-on, or bednets have effectively reduced VL infections [55,56,57,58,59]. Those strategies will vary in the logistics necessary for their large-scale application, including the re-application time of the product.

Although this study was completed in 2006, these results have only now become public. Nevertheless, our results are still relevant since the CVL National Control Program still advocates canine euthanasia. Although this measure remains important, the number of dogs euthanized reduced over time is a consequence of the positive impact of DM4 collar.

Despite the difficulties, as explained above, the use of insecticides, mainly with dogs in the risk groups, should be considered for use in public animal health and, as a consequence, the improvement of human health, due to reduce strength of infection among dogs.

The use of DM4 collars should be adopted as a prevention and control measures for CVL at the national level, to protect healthy dogs and reduce the negative impact of euthanasia, as recommended by the National Program for Control of CVL in Brazil.

## 5. Conclusions

The studied canine population exhibited a phenotypic and behavioral profile associated with L. infantum infection. Adoption of DM4 collars reduces the canine euthanasia, and the lower CVL incidence was sustained one year after the discontinued use of the collar and could be observed throughout the urban area. The estimated effectiveness was 66% and the probability of a dog’s survival reached 90% after 50 months of constant collar use. Future studies could evaluate factors associated with collar loss and monitor the anti-saliva antibody profile in dogs. 

## Figures and Tables

**Table 1:** Frequencies observed by category of the canine population, positivity to *L. infantum* infection and, results of the univariate logistic regression model.

Variable	Category	Frequency		Positivity			Univariate Logistic Model			
		N	%	N	%	P *	OR	CI (OR)		P (OR)
Sex	Female	10,775	48.1	2101	9.4	0.006	1.0			
	Male	11,629	51.9	2102	9.4		0.9	0.9	1.0	0.006
Age Interval	>10 Years Old	316	1.4	79	0.4	0.000	1.0			
	0–3 Mounths of Age	22	9.8	179	0.8		0.3	0.2	0.4	0.000
	4–11 Mounths of Age	7483	33.4	889	4.0		0.4	0.3	0.5	0.000
	1–2 Years Old	5782	25.0	1226	5.5		0.8	0.6	1.0	0.110
	3–5 Years Old	4346	19.4	1168	5.2		1.1	0.8	1.4	0.467
	6–10 Years Old	2277	10.2	662	3.0		1.2	0.9	1.6	0.134
Fur Type	Long–Curly	2218	9.9	327	1.5	0.000	1.0			
	Short–Straight	16,554	73.9	3222	14.4		1.4	1.2	1.6	0.000
	Short–Curly	1167	5.2	119	0.5		0.7	0.5	0.8	0.000
	Long–Straight	2465	11.0	535	2.4		1.6	1.4	1.9	0.000
Weight	0–20 kg	17,138	76.5	2993	13.4	0.000	1.0			
	>20 kg	5266	23.5	1210	5.4		1.4	1.3	1.5	0.000
Color Hair	White	2537	11.3	329	1.5	0.000	1.0			
	Beige	1762	7.9	358	1.6		1.7	1.5	2.0	0.000
	Graysh Brown	1624	7.2	329	1.5		1.7	1.4	2.0	0.000
	Tabby	3131	14.0	555	2.5		1.4	1.2	1.7	0.000
	Black	8535	38.1	1675	7.5		1.6	1.4	1.9	0.000
	Grey	630	2.8	128	0.6		1.7	1.4	2.1	0.000
	Brown	4185	18.7	829	3.7		1.7	1.4	1.9	0.000
Breed	Others Breeds	374	1.7	64	0.3	0.000	1.0			
	Mongrel or Mixed-Breed	8705	38.9	1862	8.3		1.3	1.0	1.7	0.048
	Akita Inu	3821	17.1	656	2.9		1.0	0.8	1.3	0.978
	Basset/Teckel/Dachshund	32	0.1	1	0.0		0.2	0.0	1.2	0.070
	Beagle	770	3.4	137	0.0		1.0	0.8	1.5	0.777
	Boxer	37	0.2	3	0.0		0.4	0.1	1.4	0.169
	Cocker Spainel	743	3.3	199	0.9		1.8	1.3	2.4	0.000
	Collie	260	1.2	56	0.2		1.3	0.9	2.0	0.162
	Dalmatian	24	0.1	3	0.0		0.7	0.2	2.4	0.560
	Dobermann	61	0.3	6	0.0		0.5	0.2	1.3	0.158
	Deutsche Dogge	142	0.6	48	0.2		2.5	1.6	3.8	0.000
	Brazilian Mastiff	28	0.1	11	0.0		3.1	1.4	7.0	0.005
	Brazilian Terrier /Fox Terrier	141	0.6	25	0.1		1.0	0.6	1.7	0.869
	Siberian Husky	292	1.3	54	0.2		1.1	0.7	1.6	0.643
	Labrador Retriever	47	0.2	14	0.1		2.1	1.0	4.1	0.038
	German Shepherd	53	0.2	7	0.0		0.7	0.3	1.7	0.476
	Pekingese/Shih-Tzu/Lhasa	813	3.6	204	0.9		1.6	1.2	2.2	0.002
	Doberman Pinscher	2753	12.3	425	1.9		0.9	0.7	1.2	0.403
	Bull Terrier	191	0.9	21	0.1		0.6	0.4	1.0	0.056
	Poodle	2776	12.4	337	1.5		0.7	0.5	0.9	0.007
	Rottweiller	324	1.4	70	0.3		1.3	0.9	1.9	0.134
	Yorkshire Terrier	17	0.1	0	0.0		0.0	-	-	0.998
Size	0–20 cm	1015	45.3	1620	7.2	0.000	1.0			
	21–49 cm	9151	40.8	1,785	8.0		1.3	1.2	1.4	0.000
	≥50 cm	3103	13.9	798	3.6		1.8	1.7	2.0	0.000
Sleep Habit at Night	Intradomicile	902	4.1	101	0.5	0.000	1.0			
	Peridomicile	21,502	95.9	4102	18.3		1.9	1.5	2.3	0.000
Vl Signal Type	Asymptomatic	21,195	94.6	3755	16.8	0.000	1.0			
	Growth Of Nails (1)	313	1.4	95	0.4		2.0	1.6	2.6	0.000
	Fall Of Hair (2)	81	0.4	26	0.1		2.2	1.4	3.5	0.001
	Weight Loss (3)	87	0.4	26	0.1		2.0	1.2	3.1	0.004
	(1) + (2)	63	0.3	27	0.1		3.5	2.1	5.7	0.000
	(1) + (3)	63	0.3	21	0.1		2.3	1.4	3.9	0.002
	(1) + (2) + (3)	102	0.5	48	0.2		4.1	2.8	6.1	0.000
	(2) + (3)	32	0.1	11	0.0		2.4	1.2	5.1	0.017
	Cutaneous Lesion (4)	182	0.8	75	0.3		3.3	2.4	4.4	0.000
	(4) + Others	286	1.3	119	0.5		3.3	2.6	4.2	0.000
Signal Presence	Absent	21,195	94.6	3755	16.8	0.000	1.0			
	Present	1.209	5.4	448	2.0		2.7	2.4	3.1	0.000
**All Categories Total**		**22,404**	**100.0**	**4203**	**18.8**					

* Note χ^2^ of Pearson 95% Significance.

**Table 2:** Factors associated with canine visceral leishmaniasis, odds ratio, and confidence interval results of the multiple logistic regression analysis.

Variables	Categories	B	S.E.	Wald	D.F.	Sig. (OR)	OR	CI 95% (OR)	
								MÍN	MÁX
Sex ^f^	Male						1		
	Female	0.09	0.04	6.27	1	0.01	1.09	1.02	1.17
Fur Type ^e^	Long–Curly						1		
	Short–Straight	−0.04	0.10	0.15	1	0.70	0.96	0.79	1.17
	Short–Curly	−0.45	0.12	14.34	1	0.00	0.64	0.50	0.80
	Long–Straight	−0.01	0.11	0.01	1	0.93	0.99	0.81	1.22
Color Fur ^g^	All Color	0.02	0.01	5.18	1	0.02	1.02	1.00	1.05
Sleep Habit at Night ^d^	Intradomicile						1		
	Peridomicile	0.47	0.11	17.88	1	0.00	1.60	1.29	1.98
Signal Presence ^c^	Absent								
	Present	0.73	0.06	126.79	1	0.00	2.07	1.82	2.34
Breed ^b^	Others Breeds						1		
	Mongrel Or Mixed-Breed	0.32	0.14	4.96	1	0.03	1.38	1.04	1.82
	Akita Inu	0.20	0.15	1.79	1	0.18	1.22	0.91	1.63
	Basset/Teckel/Dachshund	−1.82	1.03	3.12	1	0.08	0.16	0.02	1.22
	Beagle	0.08	0.17	0.21	1	0.65	1.08	0.77	1.52
	Boxer	−0.80	0.63	1.64	1	0.20	0.45	0.13	1.53
	Cocker Spainel	0.69	0.17	17.37	1	0.00	2.00	1.44	2.77
	Collie	0.30	0.21	2.00	1	0.16	1.34	0.89	2.02
	Dalmatian	−0.65	0.64	1.04	1	0.31	0.52	0.15	1.82
	Dobermann	−0.35	0.46	0.59	1	0.44	0.70	0.29	1.73
	Deutsche Dogge	0.67	0.23	8.29	1	0.00	1.95	1.24	3.07
	Brazilian Mastiff	1.06	0.43	6.20	1	0.01	2.89	1.25	6.68
	Brazilian Terrier /Fox Terrier	0.28	0.27	1.11	1	0.29	1.32	0.79	2.23
	Siberian Husky	0.10	0.21	0.25	1	0.62	1.11	0.74	1.67
	Labrador Retriever	0.78	0.36	4.79	1	0.03	2.19	1.09	4.41
	German Shepherd	−0.03	0.44	0.01	1	0.94	0.97	0.41	2.28
	Pekingese/Shih-Tzu/Lhasa	0.39	0.16	5.72	1	0.02	1.47	1.07	2.03
	Doberman Pinscher	−0.13	0.15	0.68	1	0.41	0.88	0.65	1.19
	Bull Terrier	−0.13	0.27	0.21	1	0.65	0.88	0.52	1.51
	Poodle	−0.18	0.17	1.18	1	0.28	0.83	0.60	1.16
	Rottweiller	0.39	0.20	3.85	1	0.05	1.47	1.00	2.17
	Yorkshire Terrier	−19.33	9588.26	0.00	1	1.00	0.00	0.00	
Age Interval ^a^	0–3 Mounths of Age			638.36	5	0.00	1		
	4–11 Mounths of Age	0.43	0.09	24.44	1	0.00	1.53	1.29	1.81
	1–2 Years Old	1.10	0.09	168.42	1	0.00	3.01	2.55	3.56
	3–5 Years Old	1.38	0.09	258.59	1	0.00	3.99	3.37	4.73
	6–10 Years Old	1.49	0.09	264.20	1	0.00	4.44	3.71	5.32
	>10 Years Old	1.26	0.15	66.78	1	0.00	3.53	2.61	4.78

^a^ Variable(s) entered on step 1: Age Group, ^b^ Variable(s) entered on step 2: Breed, ^c^ Variable(s) entered on step 3: Signal Presence, ^d^ Variable(s) entered on step 4: HABIT, ^e^ Variable(s) entered on step 5: Hair Type, ^f^ Variable(s) entered on step 6: Gender, ^g^ Variable(s) entered on step 7: Color, D.F.: Freedom degree, Sig.: Significance. OR: Odds Ratio.

**Table 3:** Number of months that the dogs wore the DM4 collar.

Number of Months	Dogs Number	Percent	Cumulative Percent
with Collar			
0	6096	27.2	0
6	3625	16.2	16.2
12	4076	18.2	34.4
18	3001	13.4	47.8
24	1560	7.0	54.8
30	4046	18.0	72.8
**Total**	**22,404**	**100.0**	

**Table 4:** Relative risk (RR) for CVL infection obtained by the Cox multiple model according to the independent variables.

Variables	Categories	B	Se	Wald	Sig.	RR	CI 95% (RR)	
							MIN	MAX
Age Interval ^a^	0–3 Mounths of Age					1		
	4–11 Mounths of Age	0.265	0.082	10.375	0.001	1.303	1.109	1.531
	1–2 Years Old	0.913	0.081	126.335	0.000	2.493	2.126	2.923
	3–5 Years Old	1.024	0.082	154.243	0.000	2.783	2.368	3.271
	6–10 Years Old	1.117	0.087	166.047	0.000	3.056	2.578	3.621
	>10 Years Old	0.949	0.138	47.505	0.000	2.582	1.972	3.382
Sex	Female					1		
	Male	−0.069	0.031	4.940	0.026	0.933	0.877	0.992
Weight ^e^	0–20 kg					1		
	>20 kg	0.140	0.044	9.943	0.002	1.151	1.054	1.255
Color Hair ^f^	White					1		
	Beige	0.197	0.083	5.635	0.018	1.217	1.035	1.432
	Graysh Brown	0.215	0.085	6.382	0.012	1.240	1.049	1.465
	Tabby	0.106	0.077	1.896	0.169	1.112	0.956	1.294
	Black	0.182	0.068	7.104	0.008	1.199	1.049	1.370
	Grey	0.250	0.106	5.549	0.018	1.284	1.043	1.582
	Brown	0.116	0.074	2.432	0.119	1.122	0.971	1.298
Sleep Habit at Night ^d^	Intradomicile					1		
	Peridomicile	0.440	0.102	18.538	0.000	1.553	1.271	1.898
Breed ^b^	Others Breeds					1		
	Mongrel or Mixed-Breed	0.177	0.130	1.850	0.174	1.194	0.925	1.540
	Akita Inu	0.159	0.132	1.434	0.231	1.172	0.904	1.519
	Basset/Teckel/Dachshund	−1.139	1.009	1.274	0.259	0.320	0.044	2.312
	Beagle	0.126	0.156	0.650	0.420	1.134	0.836	1.538
	Boxer	−0.574	0.591	0.941	0.332	0.564	0.177	1.795
	Cocker Spainel	0.441	0.146	9.148	0.002	1.554	1.168	2.068
	Collie	0.126	0.186	0.457	0.499	1.134	0.787	1.635
	Dalmatian	−0.293	0.592	0.246	0.620	0.746	0.234	2.379
	Dobermann	0.045	0.430	0.011	0.917	1.046	0.450	2.428
	Deutsche Dogge	0.371	0.192	3.735	0.053	1.450	0.995	2.112
	Brazilian Mastiff	0.422	0.332	1.621	0.203	1.525	0.796	2.922
	Brazilian Terrier/Fox Terrier	−0.080	0.238	0.112	0.738	0.924	0.579	1.472
	Siberian Husky	0.134	0.190	0.499	0.480	1.143	0.788	1.658
	Labrador Retriever	0.728	0.298	5.986	0.014	2.072	1.156	3.713
	German Shepherd	−0.340	0.399	0.725	0.394	0.712	0.326	1.556
	Pekingese/Shih-Tzu/Lhasa	0.141	0.144	0.957	0.328	1.152	0.868	1.529
	Doberman Pinscher	−0.111	0.138	0.649	0.421	0.895	0.682	1.173
	Bull Terrier	0.464	0.254	3.335	0.068	1.590	0.967	2.616
	Poodle	−0.143	0.143	0.992	0.319	0.867	0.655	1.148
	Rottweiller	0.181	0.175	1.074	0.300	1.198	0.851	1.688
	Yorkshire Terrier	−7.636	28.003	0.074	0.785	0.000	0.000	0.000
Signal Presence ^c^	Absent					1		
	Present	0.370	0.052	50.144	0.000	1.448	1.307	1.604

^a^ Variable (S) Entered At Step No. 1: Age, ^b^ Variable(S) Entered At Step No. 2: Breed, ^c^ Variable(S) Entered At Step No. 3: Signal, ^d^ Variable(S) Entered At Step No. 4: Habit, ^e^ Variable(S) Entered At Step No. 5: Weight, ^f^ Variable(S) Entered At Step No. 6: Color, Beginning Block No. 0, Initial Log Likelihood Function: −2 Log Likelihood: 66316.020, Beginning Block No. 1. Method = Forward Stepwise (Wald).

**Table 5:** Number of dogs according to the probability of survival obtained by the Cox model, adjusted by the independent variables.

Survival Probability	Frequency	Percent	Cumulative Percent
0	1544	6.4	6.5
0.001 to 0.249	51	0.2	6.7
0.250 to 0.499	252	1.1	7.7
0.500 to 0.749	6,017	25.1	32.8
0.750 to 1.000	16,082	67.2	100.0
**Total**	**23,948**	**100.0**	
